# Relationships between sleep duration, physical activity and body mass index in young New Zealanders: An isotemporal substitution analysis

**DOI:** 10.1371/journal.pone.0184472

**Published:** 2017-09-12

**Authors:** Borja del Pozo-Cruz, Nicholas Gant, Jesús del Pozo-Cruz, Ralph Maddison

**Affiliations:** 1 Department of Exercise Sciences, Faculty of Science, University of Auckland (Auckland, New Zealand). TAMAKI BUILDING 731 -, Level 3, Tamaki Campus Gate 1,St Johns, Auckland, New Zealand; 2 Department of Physical Education and Sports, University of Seville (Seville, Spain), Pirotecnia s/n, Seville, Spain; 3 School of Exercise and Nutrition, Faculty of Health Sciences, Deakin University (Geelong, Australia), Burwood, Victoria, Australia; Indiana University, UNITED STATES

## Abstract

**Background:**

The evidence regarding the unique effect of sedentary behaviour on obesity among children is unclear. Moreover, the effect of substituting sedentary behaviour with physical activity of different intensities on the body composition of children has received limited empirical study.

**Objective:**

To examine the mathematical effects on Body Mass Index (BMI) of substituting sedentary behaviours with physical activities of different intensities on children and youth aged 5–14 years old in New Zealand.

**Methods:**

Secondary analysis of accelerometer data from the National Survey of Children and Young People’s Physical Activity and Dietary Behaviours in New Zealand (2008/09) was conducted. A total of 1812 children and youth aged 5–24 years provided accelerometer-derived data on daily sedentary time (SB), light intensity physical activity (LPA) and moderate to vigorous physical activity (MVPA). Sleep time was assessed with a validated computerised use-of-time tool. BMI was assessed using anthropometric measurements. Multiple linear regression models were used to examine the independent associations of SB, Sleep time, LPA, and MVPA on BMI. The isotemporal substitution approach was used to ascertain the mathematical effect of substituting each of the other behaviours on BMI. Analyses were stratified by age groups.

**Results:**

SB showed a unique (inverse) association with BMI across all age groups (p<0.05) but 20–24 years (p>0.05). Similarly, MVPA was positively associated (p<0.001) across all age groups. Among age groups 5–9 years, 10–14 years and 15–19 years, the estimated impact of replacing 60 min/day of SB with the same amount of MVPA time resulted in decreased BMI for all age groups (p<0.001), ranging from -1.26 (5–9 years) to -1.43 units (15–19 years). Similar results were achieved when SB was replaced with LPA or sleeping time for children (5–19 years). In young people (age group 20–24), the impact of replacing 30 min/day of SB with MVPA resulted in an estimated -1 BMI units decrease (p<0.001).

**Conclusion:**

MVPA and SB have a unique effect on BMI. Further, substituting SB with LPA or MVPA was associated with a favourable effect on BMI across all age groups; with MVPA having the strongest association.

## Introduction

Globally, childhood obesity is a significant health issue with increasing prevalence worldwide, including New Zealand where 11% of children are obese. Obesity persists into adulthood, increasing children’s risk of chronic diseases later in life. Managing childhood obesity is therefore a public health priority.

Along with nutrition, physical activity (PA) plays a critical role in children and adolescents’ health and development [1, 2], including maintaining healthy weight [3]. There is now accumulating evidence showing an inverse relationship between moderate-to-vigorous intensity physical activity (MVPA) and body mass index (BMI) [4]. Therefore, intensity of PA seems to be a key component of programs to reduce weight among this population group. It has been also suggested that light intensity physical activity (LPA) contributes to weight loss reduction by exclusively increasing energy expenditure [5]; however it is unclear if LPA has a unique effect on obesity markers among this population group.

Sedentary behaviour (SB) is considered to be an independent cardiovascular and mental health risk factor among adults and elderly [6]. However, this relationship is yet to be confirmed among children and adolescents. A number of studies have shown a relationship between self-reported SB and adiposity markers in children and youth [7]. Nevertheless, research investigating this relationship using objectively measured SB are scarce [8]. Further, only a few studies [9–12] have taken into account the deleterious effects of SB on total energy expenditure, resulting from displacing time that could be spent in alternative energy-consuming activities. To address this issue, the isotemporal substitution paradigm has recently been proposed [13]. This paradigm assumes that activity time in a day is finite and that performing one activity involves substitution for another. Depending on the kind of activity that is replaced, the effects on health might be different. This analytical approach has been recommended for epidemiological research [13] involving PA and SB. A deeper understanding on how sleep, SB, and PA interact with BMI in children and young is highly relevant to public health professionals for identifying research-informed strategies for prevention and management of overweight and obesity. The first study to apply the isotemporal substitution paradigm to PA and SB to better understand obesity involved analysis of the US National Health And Nutrition Examination Survey (NHANES), and included 5607 participants with objectively measured physical activity. Loprinzi et al. showed that MVPA, but not LPA or SB, uniquely contributed to adiposity biomarkers among children and adolescents (aged 6–17 years) within the US. Since then several studies have applied this approach in relatively small cohorts (i.e. n<400) of children to determine the effects of substituting SB with PA on a diverse range of obesity markers [10, 11, 14]. Similar effects to those observed by Loprinzi et al., have been found in 10-yr old British children [15]. However, other studies have suggested that SB might have a harmful impact on various adiposity outcomes and cardio-metabolic biomarkers [8]. Thus, the evidence on the unique effect of SB on obesity among children is unclear.

Previous studies involving the isotemporal substitution paradigm were constrained to analysis of PA and SB activity types. However, in children, sleep is an activity that constitutes a considerable proportion of daily time and sleep duration is associated with BMI. Research has shown that screen-based sedentary time dominates the presleep period in children and young people and is associated with a later sleep onset [16]. Thus, there is likely an interaction between sleep duration, SB and PA; but studies that have analysed the unique and interactive effects of sleep duration on BMI among children and youth are rather scarce.

By applying traditional and novel regression analysis approaches (i.e. isotemporal substitution paradigm) we aimed to model the impact of sleep, SB and PA on BMI among children and youth (5–24 years) using data from a nationally representative survey from New Zealand.

## Methods

### Research design

Data from the cross-sectional National Survey of Children and Young People’s Physical Activity and Dietary Behaviours in New Zealand (2008/09) were used in this study. A representative sample of children and young people from New Zealand (n = 2,503 aged 5 to 24 years) were selected using meshblocks (i.e. a defined geographic area, varying in size from part of a city block to large areas of rural land). Within each meshblock, eligible households were identified and asked to participate in the survey. One child or young person was randomly chosen from each eligible household. Population weights were calculated for each surveyed participant and applied during all analyses. Data were collected from September 2008 to May 2009. The survey design was managed using R software for statistical computing.

Data were collected during a face-to-face home visit (computer-assisted personal interview, CAPI) and a subsequent telephone interview (computer-assisted telephone interview, CATI) conducted 7 to 14 days after the CAPI. Socio-demographic characteristics and self-reported dietary habits were collected during the face-to-face home visit. Height and weight were also measured during the home visit. Accelerometers were administered to participants to provide an objective measure of time spent in LPA, MVPA, and SB over a seven-day period. The Multi-media Activity Recall for Children and Adolescents (MARCA), a validated [17] computerised 24-hour recall time use questionnaire was used to collect information on self-reported sleeping behaviour and was administered during the CAPI and CATI (see below).

The National Survey of Children and Young People’s Physical Activity and Dietary Behaviours in New Zealand (2008/09) procedures were approved by the Multi-region Human and Disability Ethics Committee review board. Inform consent was obtained for all participants prior to any data collection.

### Variables of the study

#### Covariates (demographics)

The following demographic information was collected for all survey participants and included as covariates in statistical analysis: age, sex and ethnic group (European or other). Based on age, participants were assigned to the following age groups: 5 to 9 years; 10 to 14 years; 15 to 19 years; 20 to 24 years.

#### Body mass index

Height and weight were measured in participants’ homes using standard anthropometric methods [18] and body mass index was then calculated as weight in kilograms divided by the squared of height in meters (kg/m2).

#### Sleep duration

The Multimedia Activity Recall for Children and Adults (MARCA) [17] was utilised to obtain information on use-of-time behaviours including sleep duration. Two different versions of MARCA were used, one for children (5–17 years of age) and one for young (18–24 years of age). The MARCA is a computerised use-of-time questionnaire that takes participants through their previous day (24 hours) in segments of a minimum of five minutes duration. For discrete periods of the day (e.g., morning, afternoon and evening) the person chooses an activity from a list available and the duration of the activity, which is then recorded on the MARCA. Each participant recalled a total of three days (72 hours) of activity. During the face-to-face interview (CAPI), participants were asked to recall their activities during the previous day (24 hours). During the CATI (7 to 14 days after the CAPI), participants were asked to recall their activities during the previous two days (48 hours). In this study, only sleep duration was used for analysis.

#### Sedentary behaviour and physical activity

SB and PA were measured objectively with Actigraph accelerometers (GT1M). These devices have been validated as a measure of physical activity in children [19], adolescents, and young adults [20, 21]. The Actigraph has demonstrated acceptable validity against criterion measures with coefficients ranging from 0.50–0.89 [19, 22]. Participants were asked to wear the Actigraph in their waist during waking hours for seven consecutive days (including two weekend days). SAS version 9.2 (SAS Institute, Inc, Cary, NC) was used to reduce accelerometry data to those with ≥ 3 days of 10 h/day of monitored data using 10-sec epochs [23]. Non-wear time was defined as a sequence of ≥20 minutes of missing activity counts, with no tolerance of activity counts [24]. Freedson validated age-specific cut-points [25] were used to derive the time spent in SB, LPA, and MVPA. For each valid recorded minute, the following equation was used to convert activity counts into equivalent MET values [MET = 2.757+(0.0015*counts/min)–(0.08957*age)–(0.000038*counts/min*age)]. The obtained MET value was then converted into the corresponding level of physical activity using the defined cut-off points. For each valid participant record, total time spent in sedentary/light/moderate/vigorous-intensity activities for each valid day was averaged across the total number of days to calculate average daily time spent in each type of activity. The following cut-off points [26] for METs were used to define light-intensity, moderate-intensity and vigorous-intensity physical activity using the Actigraph accelerometer: Sedentary: <1.5 MET (0–149 counts/min); Light: 1.5 ≤ MET < 3 (150–499 counts/min); Moderate: 3 ≤ MET < 6 (500–3999 counts/min); Vigorous: ≤ 6 MET (400 or more counts/min). Total wear time was recorded and averaged per day.

### Statistical analysis

Only participants with valid accelerometer data (n = 1,812) were included in the final analysis. All statistical analyses were performed using the Statistical Package for Social Sciences (SPSS) version 22. Statistical significance was determined using a p value <0.05 throughout all analyses performed. Descriptive data are presented as Mean (95% Confidence Interval) unless otherwise stated. Multiple linear regression analysis was used to examine the associations between sleep duration, SB, LPA, MVPA and BMI. All models were adjusted for relevant covariates in each case and presented separately for each age group. Three different models were run:

#### Single-factor models

Single-factor models depict the association of each physical activity intensity category with the different outcomes of the study without mutual adjustment for other categories of activities. For instance, in a single-factor model examining the effect on BMI of 60 minutes of MVPA, the model would include MVPA and confounder variables. The exemplified model would take the form of: BMI = (b1) MVPA + (b2) confounder variables.

#### Partition model

In partition models, the coefficient for each activity intensity category represents the effect on the outcomes of the study of increasing this activity intensity category while holding other categories constant but without holding total wear time constant. Therefore, it represents the effect of ‘‘adding” an activity intensity category. Thus, all physical activity intensity categories are entered simultaneously into the model. For instance, in a partition model examining the effect on BMI of adding 60-minutes of MVPA, the model would include sedentary time, light, moderate and vigorous physical activity and other confounder variables. The exemplified model would take the form of: BMI = (b1) MVPA + (b2) LPA + (b3) sedentary time + (b4) confounder variables.

Isotemporal substitution model: Isotemporal substitution models, by definition, estimate the effect of replacing one physical activity intensity with another physical activity intensity for the same amount of time (60-minutes in our study for age groups 5 to 19 and 30-minutes for the 20–24 age group). One of the PA intensities was removed from the model, while wear time and the other PA intensities remained. Thus, coefficients represent the estimated effects on the outcomes of the study of substituting a specified intensity category for the category dropped while holding total (activity) time constant. For instance, in an isotemporal substitution model examining the effect on BMI of replacing 60-min of sedentary time with LPA, the model would include light, moderate and vigorous physical activity, total wear time, and other confounder variables. By eliminating one activity component from the model (e.g., sedentary time), the coefficient for total wear time represents the omitted activity component (sedentary time). The remaining coefficients represent the consequence of substituting 60 minutes of that activity instead of sedentary activity while holding other activity types constant. The exemplified model would take the form of: BMI = (b1) LPA + (b2) MVPA + (b4) total wear time + (b5) confounder variables.

## Results

There were no statistically significant differences in demographics or exposure variables between the different age groups of study (p>0.05). Table 1 and Table 2 present the participant demographics characteristics and accelerometer derived variables respectively according to the respective age groups.

**Table 1:** Demographics of the study sample (n = 1812).

	Mean (SEM)
Age	13.42 (0.11)
	N (%)
Sex, males (%)	51.9
Ethnicity	
Maori (indigenous)	17.6
European	8.4
Asian	11.6
Pacific	75.1
Body mass index by age group (kg/m^2^)	
	Mean (SEM)
5–9	21.02 (0.27)
10–14	20.66 (0.18)
15–19	21.01 (0.23)
20–24	20.91 (0.30)

Values are represented as mean (SEM) unless otherwise stated

**Table 2:** Accelerometry variables by age group.

Valid wear time (min/day)	Mean (SEM)
5–9	754.00 (5.10)
10–14	789.50 (5.70)
15–19	791.98 (7.04)
20–24	789.06 (9.13)
Time spent in sedentary behaviours (min/day)	
5–9	230.91 (15.13)
10–14	213.67 (11.74)
15–19	227.07 (14.02)
20–24	229.36 (17.87)
Time spent in light intensity physical activity (min/day)	
5–9	558.51 (5.63)
10–14	629.57 (9.02)
15–19	173.83 (4.61)
20–24	69.75 (4.27)
Time spent in moderate to vigorous physical activity (min/day)	
5–9	195.49 (3.51)
10–14	101.48 (2.32)
15–19	45.94 (2.22)
20–24	18.03 (1.59)
Sleep time (min/day)	
5–9	637.31 (3.56)
10–14	612.40 (3.90)
15–19	574.11 (5.23)
20–24	550.30 (7.70)

Values are represented as mean (SEM) unless otherwise stated

Table 3 presents the single behaviour, partition, and isotemporal substitution models for BMI across the different age groups. In the single behaviour models, SB was proportionally (positively) associated with BMI in all four age groups (p<0.001 for all age groups), whereas sleep time (p<0.001 for the 10–14 years and 15–19 years age groups and p<0.05 for the 5–9 years and 20–24 years age groups), LPA (p<0.001 for all age groups) and MVPA (p<0.001 for all age groups) were inversely associated with BMI.

**Table 3:** Single behaviour models for BMI among children and adolescents by age group (n = 1,812).

	Regression Coefficient (95%CI)			
Age group	Sleep Duration (min/day)	Sedentary Behaviour (min/day)	Light Intensity Physical Activity (min/day)	Moderate-to-Vigorous Physical Activity (min/day)
5–9 years	-0.010 (-0.016 to -0.004)*	0.007 (0.006 to 0.009)**	-0.007 (-0.009 to -0.006)**	-0.026 (-0.030 to -0.021)**
10–14 years	-0.016 (-0.023 to -0.010)**	0.007 (0.006 to 0.008)**	-0.006 (-0.008 to -0.005)**	-0.028 (-0.033 to -0.023)**
15–19 years	-0.018 (-0.024 to -0.012)**	0.008 (0.007 to 0.009)**	-0.008 (-0.010 to -0.006)**	-0.031 (-0.036 to -0.026)**
20–24 years	-0.016 (-0.025 to -0.007)*	0.008 (0.006 to 0.0010)**	-0.008 (-0.011 to -0.005)**	-0.038 (-0.046 to -0.031)**

*p<0.05

**p<0.001)

Covariates for models included gender, race-ethnicity and healthy diet habits (i.e. meeting fruit and vegetables daily intake guidelines).

When all behaviours were entered simultaneously in the partition models (Table 4), SB showed a unique (inverse) association with BMI across all age groups (p<0.05) but 20–24 years (p>0.05). Similarly, MVPA was positively statistically significant (p<0.001) in the partition models across all age groups.

**Table 4:** Partition models for BMI among children and adolescents by age group (n = 1,812).

	Regression Coefficient (95%CI)			
Age group	Sleep Duration (min/day)	Sedentary Behaviour (min/day)	Light Intensity Physical Activity (min/day)	Moderate-to-Vigorous Physical Activity (min/day)
5–9 years	0.003 (-0.002 to 0.007)	0.005 (0.001 to 0.009)*	0.000 (-0.003 to 0.004)	-0.016 (-0.022 to -0.010)**
10–14 years	-0.004 (-0.009 to 0.001)	0.004 (0.000 to 0.008)*	0.001 (-0.003 to 0.005)	-0.019 (-0.026 to -0.012)**
15–19 years	-0.003 (-0.009 to 0.000)	0.007 (0.003 to 0.011)*	0.003 (-0.001 to 0.007)	-0.017 (-0.025 to -0.009)**
20–24 years	0.002 (-0.006 to 0.009)	0.004 (-0.002 to 0.010)	0.002 (-0.003 to 0.008)	-0.031 (-0.043 to -0.019)**

*p<0.05

**p<0.001

Sleep time, all light intensity activity, moderate to vigorous intensity activity and covariates (gender, race-ethnicity and healthy diet habits, i.e. meeting fruit and vegetables daily intake guidelines) were simultaneously introduced in the model

Results from the isotemporal substitution models (models 6–8) were consistent across the different age groups of the study (Table 5).

**Table 5:** Isotemporal substitution models for BMI among children and adolescents by age group (n = 1,812).

	Regression Coefficient (95%CI)			
Age (5–9 years)^a^	Sleep Duration (min/day)	Sedentary Behaviour (min/day)	Light Intensity Physical Activity (min/day)	Moderate-to-Vigorous Physical Activity (min/day)
Replace Sedentary Behaviour	-0.129 (-0.444 to 0.187)	Dropped	-0.270 (-0.371 to -0.169)**	-1.255 (-1.531 to -0.980)**
Replace Light Intensity Physical Activity	-0.141 (-0.189 to 0.471)	0.270 (0.169 to 0.371)**	Dropped	-0.985 (-1.310 to -0.660)**
Replace Moderate-to-Vigorous Physical Activity	1.127 (0.658 to 1.595)**	1.255 (0.980 to 1.531)**	0.985 (0.660 to 1.310)**	Dropped
**Age (10–14 years)**^**a**^				
Replace Sedentary Behaviour	-0.487 (-0.867 to -0.107)*	Dropped	-0.172 (-0.278 to -0.066)*	-1.379 (-1.699 to -1.060)**
Replace Light Intensity Physical Activity	-0.315 (-0.702 to 0.072)	0.172 (0.066 to 0.278)*	Dropped	-1.207 (-1.585 to -0.830)**
Replace Moderate-to-Vigorous Physical Activity	0.892 (0.335 to 1.450)*	1.379 (1.060 to 1.699)**	1.207 (0.830 to 1.585)**	Dropped
**Age (15–19 years)**^**a**^				
Replace Sedentary Behaviour	-0.575 (-0.905 to -0.245)*	Dropped	-0.216 (-0.331 to -0.101)**	-1.429 (-1.767 to -1.091)**
Replace Light Intensity Physical Activity	-0.358 (-0.701 to -0.016)*	0.216 (0.101 to 0.331)**	Dropped	-1.213 (-1.521 to -0.762)**
Replace Moderate-to-Vigorous Physical Activity	0.855 (0.320 to 1.389)*	1.429 (1.009 to 1.767)**	1.213 (0.807 to 1.619)**	Dropped
**Age (20–24 years)**^**b**^				
Replace Sedentary Behaviour	-0.066 (-0.320 to 0.189)	Dropped	-0.044 (-0.133 to 0.045)	-1.047 (-1.332 to -0.763)**
Replace Light Intensity Physical Activity	-0.021 (-0.289to 0.246)	0.044 (-0.045 to 0.133)	Dropped	-1.003 (-1.344 to -0.663)**
Replace Moderate-to-Vigorous Physical Activity	0.982 (0.545 to 1.419)**	1.047 (0.763 to 1.332)**	1.003 (0.663 to 1.334)**	Dropped

^a^Prior to the regression models, all behaviour variables were divided by a constant of 60 so that unit increase in the behaviour represented an increase of 60min/day within the given behaviour

^b^Prior to the regression models, all behaviour variables were divided by a constant of 30 so that unit increase in the behaviour represented an increase of 30min/day within the given behaviour

*p<0.05

**p<0.001)

Covariates for models included gender, race-ethnicity, total wear time (sleeping time + sedentary behaviour + light intensity physical activity + moderate to vigorous physical activity) and healthy diet habits (i.e. meeting fruit and vegetables daily intake guidelines).

For model “Replace Moderate-to-Vigorous Physical Activity”, Sleeping behaviour, sedentary behaviour, light intensity physical activity and covariates were entered in the model (moderate to vigorous physical activity dropped)

For model “Replace Light Intensity Physical Activity”, Sleeping behaviour, sedentary behaviour, moderate to vigorous physical activity and covariates were entered in the model (light intensity physical activity dropped

For model “Replace Sedentary Behaviour”, Sleeping behaviour, light intensity physical activity, moderate to vigorous physical activity and covariates were entered in the model (sedentary behaviour dropped)

### Replacing SB

In age groups 5–9 years, 10–14 years and 15–19 years, the impact of replacing 60 min/day of SB with the same amount of MVPA time resulted in an estimated decrease in BMI across the different age groups (p<0.001), ranging from -1.26 (5–9 years) to -1.43 units (15–19 years). Similar results were achieved when replacing SB with LPA or sleeping time among age groups 5–19 years. In the age group 20–24, the impact of replacing 30 min/day of SB with MVPA resulted in an estimated decrease of 1 BMI unit (p<0.001). However, replacing SB with LPA or sleeping time resulted in no significant association with BMI in young people.

### Replacing LPA

Replacing LPA with SB resulted in an estimated increase in BMI of 0.27 kg/m^2^ (p<0.001) for the 5–9 years age group and 0.17 and 0.21, p<0.05 for the 10–14 years and 15–19 years age groups respectively) of BMI. In contrast, replacing LPA with MVPA resulted in an estimated decrease (-0.98 BMI units, p<0.001 for the 5–9 years age group and 120 BMI units, p<0.05 for both, the 10–14 years and the 15–19 years age groups respectively) in BMI. In the age group 20–24, a significant association with BMI was found (-1 BMI units, p<0.001) only when LPA was replaced with MVPA.

### Replacing MVPA

In5-9 years, 10–14 years and 15–19 years age groups, replacing 60 min/day of MVPA with SB was associated with an estimated increase in BMI (p<0.001), ranging from 1.25 units in the 5–9 years group to 1.45 units in the 15–19 years group. Similarly, replacing MVPA with LPA or sleeping time resulted in a systematic increase in BMI across the different age groups. This was consistent with the results in the 20–24 years age group, where replacing 30 min/day of MVPA with any of the other three behaviours (sleeping time, SB or LPA) was associated with a decrease in BMI (p<0.001).

## Discussion

In this paper, we used nationally representative data and a combination of traditional and novel regression analytical approaches to model the relationship between sleep time, SB, PA and BMI in children and youth. After controlling for each of the respective categories of physical activity, the main findings were that MVPA and SB time were significantly associated with BMI. Also, replacing SB with PA of any intensity (i.e. LPA or MVPA) mathematically reduced BMI. Our study expands the literature in this area in the following ways: 1) we extended age groups from previous studies involving nationally representative samples to include adolescents and young adults. Using these data we were able to conduct subgroup analysis by age; 2) we used objective measures of PA, SB and BMI; 3) we included sleep duration, an important component of the day that has potential to influence BMI.

Supporting results from smaller studies [10, 11, 14], our isotemporal substitution models revealed unambiguous cross-sectional reductions of BMI after SB was replaced with MVPA, and replacing SB with MVPA produced greater BMI reductions compared to LPA. Interestingly, our results suggest that when SB is replaced with LPA a reduction in BMI could be achieved. These results contrast with those found in smaller studies by Sardinha et al. in 386 children age 9–12 years old [12] and Leppanen et al. in 307 children age 4 years old [14] where this effect was not reported. Not surprisingly, replacing MVPA with LPA results in adverse BMI outcomes in our study sample. In sum, our findings suggest that promoting MVPA and reducing SB is associated with lower body mass and highlighted the potential positive effects that LPA on BMI.

Not surprisingly, in single models MVPA and LPA were associated with lower BMI across all age groups. This supports traditional thinking that PA, regardless of intensity, is associated with increased energy expenditure and can contribute to energy related behaviours such as obesity. On the other hand, greater SB was associated with higher BMI among the population sample. However, after controlling for each of the other behaviours (partition models) only SB and MVPA remained statistically significant, demonstrating the unique effect of these behaviours on BMI. Our results align with previous work suggesting that higher-intensity PA is more strongly associated with lower BMI among 10-year old British children [15].

Contrary to previous work [12, 15] our data suggest that SB was associated with BMI across all ages under study. The context in which SB occurs may partly explain the former observations. A systematic review concluded that watching television for 2 h per day was associated with unfavourable body composition and reduced fitness among children and youth [27]. Watching TV was high (>2h/day) among our sample [23] and TV watching may also increase energy intake among children and adolescents [25]. However, longitudinal studies are necessary to demonstrate whether SB contributes independently of MVPA to obesity among children and youth.

In recent time, sleep has been considered to have an impact on weight status among children and adolescents [28–30]. It has thus been highlighted as a potential target in obesity interventions [31]. However, our data do not support an effect of sleep time on BMI in our sample. More research is needed to clarify the impact of sleeping time, SB and PA on obesity among children and youth.

Strengths of the study include the large nationally representative sample, objective measures of PA and SB, the different age-groups, and inclusion of time use behaviour, as well as the novel analytical approach (isotemporal substitution modelling). Nevertheless, the following limitations need to be considered when interpreting these findings. First, the cross-sectional nature of the research does not allow causal inferences to be drawn. Second, there are some inherent issues with SB and PA being derived from accelerometers, such as the use of <100 counts/minute as a threshold to determine sedentary activities or epoch length that may impact the generalization of the results [26]. Similarly, the use of self-reported measures to obtain data on sleeping behaviour of participants is associated with issues of recall bias. Longitudinal designs are necessary to overcome some of the research-design inherent limitations of this study and confirm the results reported here.

## Conclusion

In conclusion, this secondary data analysis of children and adolescents from New Zealand showed that MVPA and SB have uniques effect on body composition. Substituting SB with LPA or MVPA is associated with a favourable effect on BMI across all age groups, with MVPA having the strongest association. These findings support the potential value of reducing SB time and possible substitution with PA in children and youth to control weight. More research is required to determine the potential effects of LPA to control weight in this population group.

## Ethics approval and consent to participate

This national survey was voluntary and relied on the goodwill of participants. Parental consent was obtained for participants 15 years and under, while older participants provided consent themselves. Data were collected directly from children aged 10 years and older, whereas parents provided proxy responses for children 9 years and under. The University of Auckland Human Participants Ethics Committee approved the study.

## Abbreviations

SB: Sedentary Behaviour
LPA: Light Physical Activity
MVPA: Moderate-to- Vigorous Physical Activity
BMI: Body Mass Index
NHANES: National Health and Nutrition Examination Survey
PA: Physical Activity
CAPI: Computer-Assisted Personal Interview
CATI: Computer-Assisted Telephone Interview
MARCA: Multi-Media Activity Recall for Children and Adolescent
